# Diverticulosis and Diverticulitis: Epidemiology, Pathophysiology, and Current Treatment Trends

**DOI:** 10.7759/cureus.43158

**Published:** 2023-08-08

**Authors:** Mohit Bhatia, Aastha Mattoo

**Affiliations:** 1 Surgery Department, Princess Royal University Hospital, King's College, Orpington, GBR; 2 Emergency Department, Princess Royal University Hospital, King's College, Orpington, GBR

**Keywords:** hartmann procedure, acute surgical abdomen, acute diverticulitis, diverticula, diverticulosis

## Abstract

Diverticular disease is a common surgical condition, especially in the Western world. Its existence is well known in Asian countries as well; however, its impact on Asian health care is not the same as that in Western countries. Diverticular disease has a variable presentation, and its implications can be challenging to manage both for the patient and the medical professionals. Diet and lifestyle are commonly associated with its etiology. In Western countries, much attention is given to diverticular disease, and with the acceptance of colonoscopy as a surveillance investigation, a greater number of people are diagnosed with diverticular disease at an early stage and overall. In acute presentations, a CT scan of the abdomen remains the investigation of choice. The most common presentation of diverticular disease is pain in the abdomen or a change in bowel habits. In most cases, diverticular disease is treated with medical intervention; however, in cases associated with severe complications or advanced stages, surgical modality remains the primary treatment.

## Introduction and background

Diverticulosis refers to an outpouching of colonic mucosa and submucosa. It commonly occurs around the penetrating blood vessels [1]. It can occur in any segment of the bowels; however, the sigmoid colon is the most affected segment [2]. In the past two decades, diverticular disease is one of the most commonly diagnosed bowel disorders. It is seen across the globe; however, its incidence is high, especially in Western countries [3]. It is estimated that in the United Kingdom alone, 50% of the population above 50 years of age is affected by diverticular disease [4]. Diverticular disease was believed to be associated with increasing age; however, in recent studies, its prevalence in younger populations has increased [5]. In about 10-25% of patients with diverticulosis, the disease progresses and leads to diverticulitis [6].

Many theories have been suggested for the different etiologies of diverticular disease, and the most common cause is believed to be a low-fiber diet. It is believed that diets low in fibers are associated with increased intra-colonic pressure, which leads to diverticula formation [7].

## Review

Epidemiology

The incidence of diverticular disease has increased in the past few decades [8]. While the prevalence of diverticular disease has always been high in the Western world compared to that in Asian countries, the prevalence in Asian populations has recently increased with an incidence rate of 0.5% being reported [9,10]. A study by Painter et al. suggested that an increase in intra-colonic pressure is responsible for the development of diverticular disease [11]. With the global adoption of Western diets and rampant immigration, there is less disparity among populations worldwide [12].

Wheat et al. concluded that white women undergo more hospitalizations due to diverticular disease [13]. While earlier studies showed that men were affected more, recent studies have reported that the incidence of diverticular disease is also getting high in women. Data from the United Kingdom revealed high hospital admissions among females due to diverticular disease [14]. A study reflected on the protective role of testosterone on the colonic walls in preventing diverticula formation [15]. A low-fiber diet is considered to be a major contributing factor in the development of the disease; however, the exact mechanism is not clear [16].

In Western countries, the left colon is found to be more affected compared to the right-sided colon involvement, which is higher in Asian populations [17]. The difference in locations of the diverticula has been a much-debated subject. A British study by Golder et al. concluded with the help of enemas that the Black African population has a larger involvement in the proximal colon than the white population [18]. Colonoscopy-driven studies have shown similar prevalence rates of diverticular disease in Lebanon compared to America [19].

Yamamichi et al. reported that the incidence of diverticular disease increased from 13% (1990-2000) to 23.9% (2000-2010) [20]. Although the predominance of right-sided diverticular disease in the Asian population was believed to be of congenital origin, many studies have challenged this theory [19]. A genetic study showed the involvement of some specific genes such as TNFSF15 single nucleotide polymorphism (SNP) in the development of diverticulitis [21]. Some authors reflected upon the degradation of the myenteric plexus and Cajal cells as a cause of increasing intra-colonic pressure and subsequent diverticular disease [22].

Presentation

Diverticular disease can have variable clinical presentations. Most often it presents as abdominal pain or change in bowel habits and rectal bleeding. Its clinical picture can at times mimic inflammatory bowel disease or bowel malignancy. Its common sequelae include diverticulitis, perforation, and peri-colic abscess/phlegmon.

Pain during diverticular bleed is classified as non-specific lower abdomen pain and is associated with raised inflammatory markers and temperature in diverticulitis. It is estimated that 4-15% of patients with diverticular disease have episodes of diverticulitis [23]. In 2010, white populations had a high prevalence of diverticulitis (75.5 of 100,000). Diverticulitis occurs more in males younger than 50 years of age [24]. Diverticular abscess is seen in around 17% of the patients with the diverticular disease [25]. More serious complications include peritonitis, fistula formation, and bowel stricture [26]. Only 1-2% of patients have diverticular perforative peritonitis with high mortality rates [27].

Wong et al. concluded that 38% of the patients have right-sided diverticulitis, and 49% have left-sided diverticulitis [28]. The incidence of complications is believed to be associated more with left-sided diverticulitis. The American Society of Colon and Rectal Surgery (ASCR) postulated that decisions for surgical intervention should be based on the patient’s overall health status and recurrent episodes should not be the sole criteria. They also pitched a laparoscopic approach for better outcomes [29].

Complicated Course

The complications of diverticular disease include abscess, fistula, and bowel strictures. Small or localized collections can be managed with intravenous antibiotics and depending on the available resources, intervention radiology can also be used to manage such cases [30].

Diagnosis

With the advent of modern radiological tools, early and accurate diagnosis of diverticular disease has become possible. Ultrasonography, CT scan, and barium studies have all been used for its diagnosis. However, all of them have their limitations.

Ultrasound: It is widely available and inexpensive, and therefore can be easily used. However, its main limitation is that it is an overtly operator-dependent study and, therefore, not a very accurate investigation. However, it is considered specific and highly sensitive for uncomplicated diverticular disease [31].

CT scan: It is considered a more specific and detailed study. It can also be used for reviewing the patient's progress to treatment, and with serial scans further treatment planning can also be conducted. It can also be used for therapeutic purposes such as drainage of abscesses and reducing the need for surgical intervention. It also helps in detecting diverticular bleeding. Its main limitations are radiation exposure and the scarcity of proper healthcare infrastructure, especially in developing countries [31].

MRI: Its use is popular in certain centers due to the absence of radiation exposure. However, the non-availability, especially in acute settings in developing countries, is a limitation [32].

Endoscopic evaluation: Colonoscopy or flexible sigmoidoscopy helps in direct visualization, as well as tissue diagnosis. However, in acute attacks, its role is controversial and the procedure is avoided. There has been a debate about the use of colonoscopy routinely after the resolution of an acute attack of diverticulitis. Some authors, in fact, recommended the use of flexible sigmoidoscopy as an alternative as the sigmoid colon is the most common site for diverticular disease [33].

Many classifications are based on CT results. However, with time, many modifications have been made to the existing ones, which impact the treatment guidelines. When used in combination with clinical and laboratory investigations, it helps in understanding the disease course and predicting the outcomes of the disease [34, 35]. Classifications for prognosis and treatment are shown in Table 1 and Table 2.

**Table 1:** Buckley Classification Sourced from [34]

Class	CT findings
Mild disease	Bowel wall thickening, fat stranding
Moderate disease	Bowel wall thickness >3 mm, phlegmon/small abscess
Severe disease	Bowel wall thickening >5 mm, perforation with subdiaphragmatic free air, abscess >5 mm

**Table 2:** Hinchey's Classification Sourced from [35]

Class	CT findings
Stage I	Pericolic abscess/phlegmon
Stage II	Pelvic, intra-abdominal, or retroperitoneal abscess
Stage III	Purulent peritonitis
Stage IV	Faecal peritonitis

Treatment

Uncomplicated Disease

The majority of the patients have an uncomplicated course of diverticular disease, which is successfully treated with antibiotics, bowel rest, and analgesia. The prescribed antibiotics should cover both aerobic and anaerobic bacteria [36]. All the hospitals have their own set of guidelines for antibiotic use. Some European studies have concluded that in mild cases of diverticulitis, antibiotics are not always required [37].

The American Gastroenterology Association (AGA) now recommends antibiotic use for the treatment of mild cases to slow down the progression of the disease and to reduce the complication rates [38]. The use of a low-fiber diet in managing diverticulitis remains controversial. Some studies have suggested that it helps in relieving symptoms; however, there is not much evidence to support it [39]. Studies have advocated using medicines like mesalamine, probiotics, and rifaximin in treating diverticulitis. A consensus has not been reached over the use of these medicines because of inadequate data and evidence [40].

Surgery/Interventions

CT-guided drainage: Collections, which are amenable to CT-guided drainage, are done to avoid surgery in a certain number of cases. However, this requires a proper setting and adequate expertise. Approximately 15-20% of patients have abscess reported on CT scan with acute episodes of diverticulitis [41]. It is believed that antibiotic therapy is highly effective in resolving diverticular abscess and has an associated failure rate of 20% [42].

It is generally considered that the size of the abscess may be a limiting factor for antibiotics to completely resolve the collection. The collection size of 4 to 5 cm is considered amenable for managing with antibiotics +/- percutaneous drainage [43]. A study by Ambrosetti et al. concluded that 54% of patients treated with percutaneous drainage during the initial episode did not require surgical intervention [44].

Surgery is required for unwell patients or those with advanced disease, with the most performed surgical intervention in complicated diverticular disease being Hartmann’s procedure. Diversion colostomy allows the inflammation/infective process to settle down and thereby reduces sepsis [45]. Scandinavian studies support the use of laparoscopic lavage in managing perforated diverticular disease, with a decreased rate of stoma formation being reported [46].

Sartelli et al. postulated primary anastomosis +/- diversion stoma for Hinchey’s III and recommended primary anastomosis +/- diverting stoma +/- damage control surgery for Hinchey’s IV. Some other authors have also recommended surgical options in patients with peritonitis secondary to diverticular perforations [47] (Table 3).

**Table 3:** Recommendations for treatment of perforated diverticulitis

Authors	Year	Hinchey’s III	Hinchey’s IV
Sartelli et al. [47]	2016	Primary anastomosis +/- stoma	Primary anastomosis +/- stoma +/- damage control surgery
Binda et al. [48]	2015	Primary anastomosis +/- stoma	Hartmann’s procedure
Agresta et al. [49]	2012	Laparoscopic lavage only	Primary anastomosis +/- stoma
Fozard et al. [50]	2011	Laparoscopic lavage	Laparoscopic lavage

The LOLA trial was terminated in 2013 due to increased failure rates with the laparoscopic lavage, and since then newer trials have not recommended laparoscopic lavage as a definitive treatment [51]. Studies have also indicated better results of resection with ileostomy compared to Hartmann’s procedure. However, additional data and consensus will help in taking things forward. Surgery in elective settings has better outcomes and requires planning and optimization. The main aim is to stabilize the patient, decrease morbidity, and improve the quality of life for the patient [52].

## Conclusions

Diverticular disease is a common surgical condition that presents differently in patients and, at present, is managed with different protocols worldwide. More evidence-based studies will help in understanding its pathogenesis better, which can help in forming more precise guidelines for effective treatment. Further research in medical management will also help in decreasing the load on health infrastructure. The endoscopic evaluation also helps in the exclusion of sinister pathology, which may mimic features of diverticular disease. However, it remains a challenge despite getting much attention worldwide.
